# Efficient Nonenzymatic Electrochemical Detection of Glucose Using CuO Nanoparticles@CNT‐Wrapped Graphene Oxide Composite Electrode

**DOI:** 10.1002/ansa.70019

**Published:** 2025-05-10

**Authors:** Amina Khalid, Rizwan Shoukat, Abid Ali, Salih Akyürekli, Arfaa Sajid, Muhammad Adeel Asghar, Qaisar Manzoor, Arif Nazir, Norah Alsadun, Amel Y. Ahmed

**Affiliations:** ^1^ Department of Chemistry The University of Lahore Lahore Pakistan; ^2^ Department of Mechanical Chemical and Materials Engineering University of Cagliari Cagliari Italy; ^3^ Innovative Technologies Application and Research Center (YETEM) Süleyman Demirel University Isparta Türkiye; ^4^ Department of Chemistry Faculty of Science King Faisal University Al‐Ahsa Saudi Arabia

**Keywords:** carbon, copper oxide, electrode, glucose Sensing, nanohybrid

## Abstract

A highly sensitive and stable nonenzymatic glucose biosensor has been developed via composite materials composed of CuO and graphene oxide (GO)/carbon nanotube (CNT) nanohybrid (CuO/GO/CNTs). Copper oxide nanoparticle(NP)‐modified CNTs were stacked via graphene sheets and synthesized through hydrothermal method, providing a larger surface area with boosted catalytic activity for efficient mass and electron passage, respectively. Scanning electron microscopy (SEM) and energy‐dispersive x‐ray (EDX) spectroscopy have been used to investigate the morphology and composition of as‐prepared nanohybrids, whereas x‐ray diffraction (XRD) patterns provide information about the crystal structure and lattice parameters. Fabricated nanohybrid was used as electrode material to develop the nonenzymatic glucose biosensor, which exhibited better performance with a linear dynamic range from 0.06 to 0.74 mM, a high sensitivity of 328 mA mM^−1^ cm^−2^ and a low detection limit of up to 0.033 mM with a fast response time of 2 s. Although the stability and reusability of the fabricated electrode have been tested. The limit of detection was determined by using the traditional formula LOD = (SNR × *σ*)/Slope. The outcomes recommend the synthesized novel structured nanohybrid as a promising material possessing significant impact for flexible and wearable biosensing applications.

## 1 Introduction

Accurate and reliable detection of blood glucose concentration is significant to control diabetes mellitus. Moreover, glucose detection is also important for environmental applications and food industries [1, 2, 3, 4]. Several methods have been developed for glucose sensing, like chromatography [5], photoacoustic resonance [6], optical methods [7], Raman spectroscopy [8, 9] electro‐chemiluminescence [10], calorimetric techniques [11], spectroscopic techniques [12] and electrochemical methods. Among these methods, electrochemical sensing offers a facile approach, fast current signal response, low detection limit, high sensitivity and facile fabrication. Additionally, electrochemical sensors can detect biomolecules without damaging their structural properties [13, 14, 15, 16]. Due to the difficult immobilization procedure and increased sensitivity towards temperature, pH and humidity affecting shelf life and performance of glucose detection strips associated with enzymatic glucose sensors, research interest has grown in developing an interference‐free, sensitive and affordable nonenzymatic glucose sensor.

A significant amount of research has been performed on the utilization of transition metals and their oxides in the development of highly efficient electrochemical biosensors. Transition metals and their composites possess excellent catalytic activity, superior surface properties, high stability in harsh chemical/electrochemical environments, non‐toxicity, biocompatibility, multiple oxidation states, fast electron transfer, high sensitivity and selectivity. Transition metal oxide nanoparticles (NPs) have received increasing attention because of their high stability, high electrical conductivity, lower production cost, easy availability and superior electrocatalytic performance towards the oxidation of glucose. You et al. prepared 2.6% and 4.5% CuO NPs in graphite‐like carbon film by using co‐sputtering method and reported higher electrooxidation activity of higher concentration (4.5%) film towards glucose [17]. Batchelor–McAuley and group [18] showed that CuO NPs played a significant role instead of MWCNTs (without metal NP catalyst) for oxidation of glucose in the basic medium by cyclic voltammetry, further proving the importance of CuO NPs in nonenzymatic glucose detection. In another research, multi‐walled carbon nanotubes (CNTs) were functionalized by Fe_3_O_4_ NPs prepared by mixing and calcination of FeCl_3_ and FeCl_2_ in hydroxide ion (OH^1−^) solution for 2 h at 300°C. The as‐prepared Fe_3_O_4_/MWCNT sensor demonstrated enhanced sensitivity of 238.69 µA mM^−1^ cm^−2^ and LOD of 15 µM with a linear range of 0.5–7 mM [19].

The use of carbon‐based materials such as CNTs and graphene and the incorporation of NPs in carbon‐based materials have been extensively explored for electrochemical biosensors in recent decades due to significant characteristics possessed by carbon nanoparticles (CNs) that include their good electrochemical activity [20, 21], large surface area [22], ease of functionalization [23, 24] and biocompatibility [25] with flexible substrate for wearable electronic textiles. The integration of CNTs in graphene can create a physical barrier between graphene layers and inhibit the aggregation of graphene sheets due to Van der Waal and strong π–π interactions, thereby increasing the surface area. Furthermore, such hybrids have enhanced optical, mechanical and electronic properties [26, 27]. For instance, a glucose biosensor was prepared by electrodeposition of Ni–Co nanostructures on reduced graphene oxide (rGO)‐modified glassy carbon electrode (GCE). This sensor depicted a low limit of detection (3.8 µM) and a linear response range of 10 µM–2.65 mM [28]. Chen et al. reported a Pt–Ni–rGO hybrid modified GCE by using the galvanic replacement method for glucose sensing, which exhibited higher sensitivity, selectivity, LOD of 2.0 µM and good stability [29]. Utilizing microwave‐assisted synthesis, Tian et al. prepared an electrode by decorating sulphur‐doped graphene (SG) with CuO NPs for glucose detection with LOD 80 µM [30]. Sheza et al. synthesized a facile electrode based on Co‐POM‐CNT fibre (Wells–Dawson‐type cobalt polyoxometalate) for electrochemical detection of glucose and ascorbic acid (AA). The reported electrode displayed efficient electro‐oxidation of glucose (response time ∼4 s) with superior sensitivity of 1000 µAmM^−1^ cm^−2^ wide linear range of 23 mM with low LOD of 0.4 µM [31].

Herein this work, we have reported a facile, economical and efficient approach to fabricate a novel structure nanohybrid composed of copper oxide–modified CNTs wrapped by graphene oxide (GO) sheets, as shown in the schematic illustration. Metal oxides offer several advantages, including cost‐effectiveness, controllable synthesis, functional biocompatibility, chemical stability and improved electron transfer kinetics. Advances in nanofabrication have led to the development of numerous metal oxide NPs as sensing materials for glucose and acetaminophen detection. In this study, CuO is investigated primarily due to its low cost, environmentally friendly properties, and its widespread use in solar energy conversion and gas sensing applications. As a p‐type semiconductor, CuO is also utilized in various applications, such as anode materials for lithium‐ion batteries, supercapacitors and biosensors [32, 33, 34]. GO is preferred due to its rich oxygen functional groups, which enhance the dispersion and interaction with CuO NPs and CNTs, facilitating improved electron transfer and catalytic activity. Although rGO offers higher conductivity, the choice of GO was likely driven by its superior structural integration and functionalization potential for efficient glucose detection. Fabricated nanohybrid coated over the surface of a fibrous structure substrate (CNTs fibre) and used as electrode material for the nonenzymatic glucose sensing. The flexible biosensor showed excellent performance due to the synergistic effect of CuO NP‐based nanohybrid and CNTs fibre that imparted boosted catalytic active sites and enhanced charge transport rate, respectively.

Our research addresses the critical need for highly sensitive and stable nonenzymatic glucose sensors by developing a CuO NPs@CNTs‐wrapped GO composite electrode. Unlike conventional sensors, our approach enhances electron transfer, increases active surface area and improves stability, offering superior performance in glucose detection.

**SCHEME 1:**
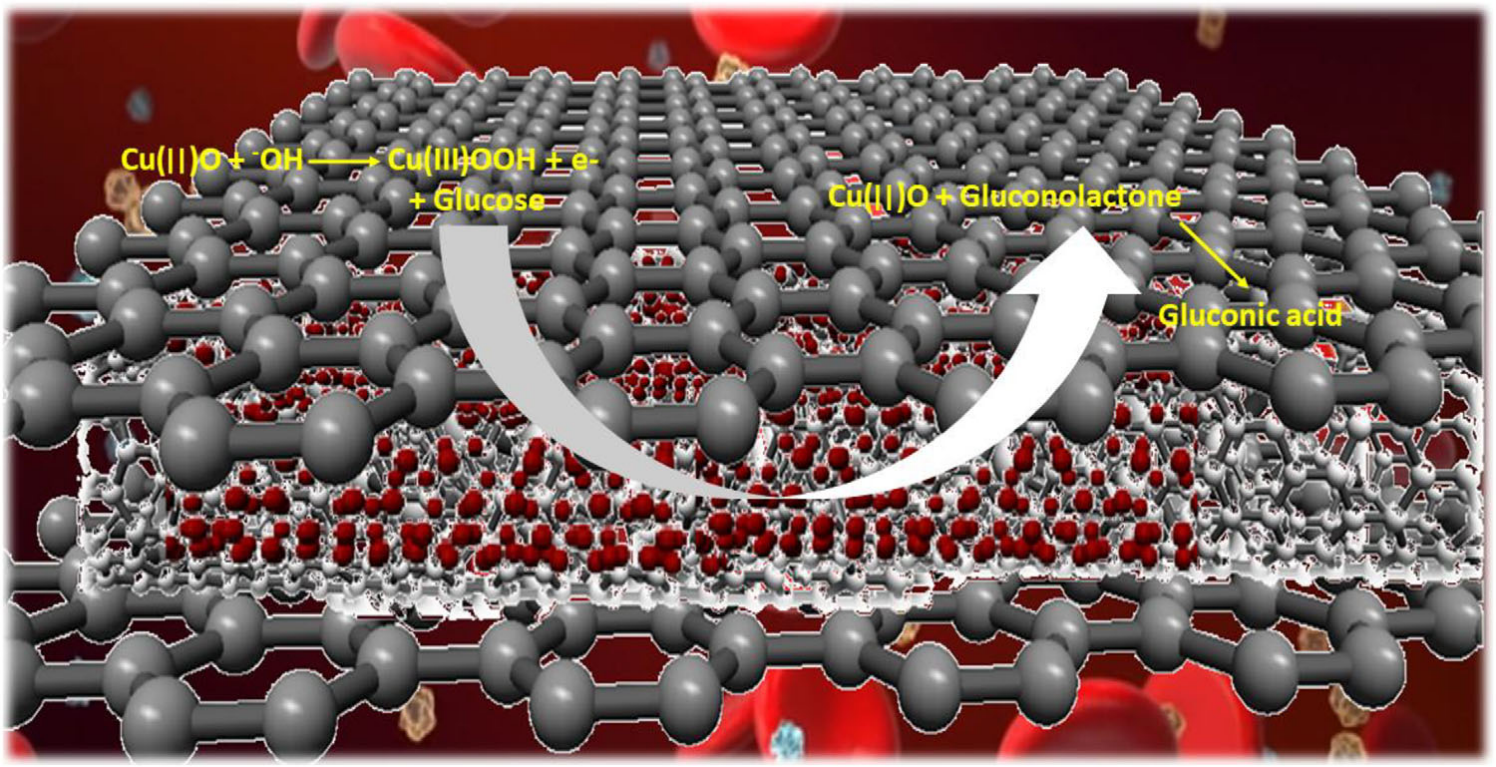
Schematic illustration for the electrochemical oxidation of glucose over the graphene‐based nanohybrid.

## 2 Experimental Section

### 1 Materials

CNTs fibres were synthesized by the wet chemical process and used as received. Ethanol (C_2_H_5_OH) and potassium hydroxide (KOH) were purchased from Merck, whereas glucose (anhydrous), hydrazine, Nafion, CuSO_4_·5H_2_O and H_2_SO_4_ were purchased from Sigma‐Aldrich. Analytical grade chemicals were used as received without further purification.

### 2 Synthesis of CuO@CNTs/GO Composite

GO was prepared by Hummer's method using graphite powder as a precursor. CNTs (multi‐walled) powder was functionalized using a mixture of HNO_3_ and H_2_SO_4_ followed by washing with distilled water three to four times, resulting in a hydrophilic CNTs suspension. CNT suspension per 3 mL of H_2_O mL of 5 mg and 16 mL of propanol were mixed in a beaker and sonicated for 30 min. Then 20 mL of 0.01 M CuSO_4_ solution was added and stirred for 30 min on a hot plate at 500 rpm. Hydrazine of 10 mL was added dropwise to the solution as a reducing agent, followed by stirring for 30 min. After the colour change, 2 mL GO suspension was mixed with the above solution, and the volume was made up to 80 mL with distilled water. The prepared suspension was transferred into a Teflon‐lined stainless steel autoclave and heated in an oven at 120°C for 8 h. After hydrothermal treatment, the resulting composite was allowed to cool down at room temperature. After that, it was washed two or three times with distilled water to remove residual impurities. Finally, the composite was dried in an oven at 60°C.

### 3 Electrode Fabrication

CNTs fibre having a diameter of ∼150 µm and a length of 2 cm was washed thoroughly with ethanol and distilled water. Then fibre was functionalized by placing it in 50% H_2_SO_4_ for a few minutes. A glass slide, washed with ethanol and dried in an oven, was used as a support for CNTs fibre. CuO/GO/CNT porous composite of 2 mg was taken in a sample vial. A thick slurry of the composite was prepared with a mixing of 5 mg of sample (CuO@CNTs/GO) in 50 µL of Nafion binder solution under sonication for 30 min. A simple glass slide was used to provide support for CNTs fibre, which was coated with the thick slurry of composite by the drop‐casting method. Modified CNTs fibre was dried in an oven, and indium metal was applied on one end of the fibre to make the electrode connection. Scheme 1 shows the schematic illustration of the fabricated electrode.

### 4 Electrochemical Studies

Electrochemical studies were performed by using a three‐electrode system using a portable potentiostat. Modified CNTs fibre was employed as a working electrode, whereas Pt sheet wire and Ag/AgCl electrodes were used as counter electrodes and reference electrodes, respectively. 0.1 M KOH solution was used as an electrolyte to provide the basic environment throughout experiments. Cyclic voltammetry was performed in a potential window of 0–0.7 V with respect to Ag/AgCl electrodes, whereas amperometric measurements were performed within the faradaic region in a steady‐state condition at a fixed potential of 0.5 V versus Ag/AgCl as a reference electrode. All the measurements were conducted three times at room temperature (25°C), and the mean value was employed for analysis.

## 2 Results and Discussion

### 2.1 Material Characterization

**FIGURE 1:**
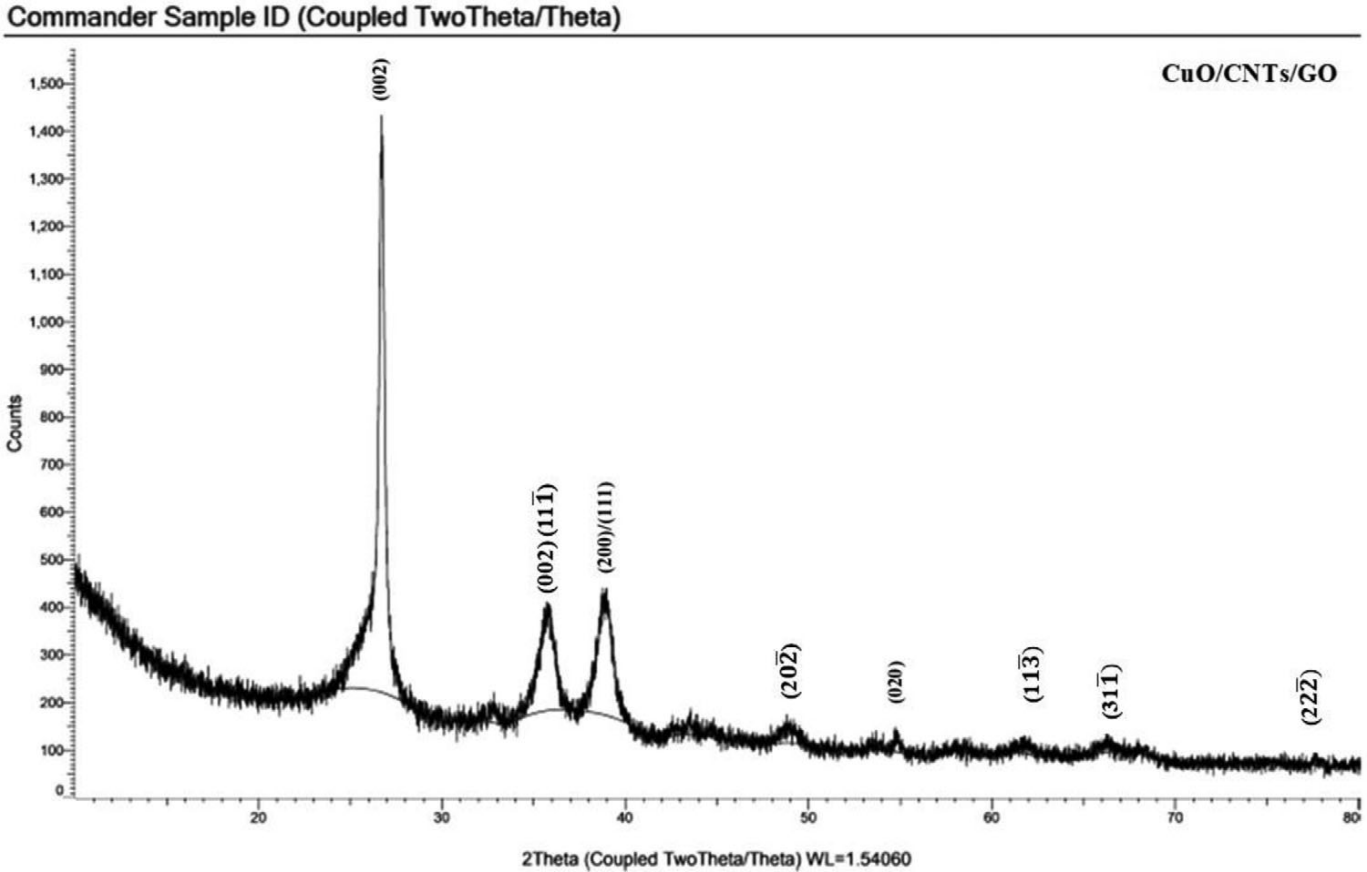
XRD pattern of CuO@CNTs/GO nanohybrid.

X‐ray diffraction (XRD) pattern of as prepared nanohybrid (CuO@CNTs/GO) composite is shown in Figure 1. The most prominent, sharp and intense peak is the (0 0 2) peak, which appears around 26° and represents the large interlayer spacing characteristics of graphene layers in nanotubes [35]. The diffraction peaks present at 36°, 39°, 49°, 55°, 62°, 66.5° and 78° could be assigned to (0 0 2), (**(1** **1** $\bar{1}$ **)**.), (2 0 0), (1 1 1), (**(2** **0** $\bar{2}$ **)**.), (0 2 0), (**(1** **1** $\bar{3}$ **)**.), (**(3** **1** $\bar{1}$ **)**.) and (**(2** **2** $\bar{2}$ **)**.) reflection planes, respectively. The planes of (0 0 2)/(**(1** **1** $\bar{1}$ **)**.) and (2 0 0) (1 1 1) are characteristics of the pure monoclinic crystal structure of CuO [36]. No other peak was observed, representing the high purity of the prepared composite. The obtained results are in good agreement with the JCPDS (48‐1548) data of CuO [37].

**FIGURE 2:**
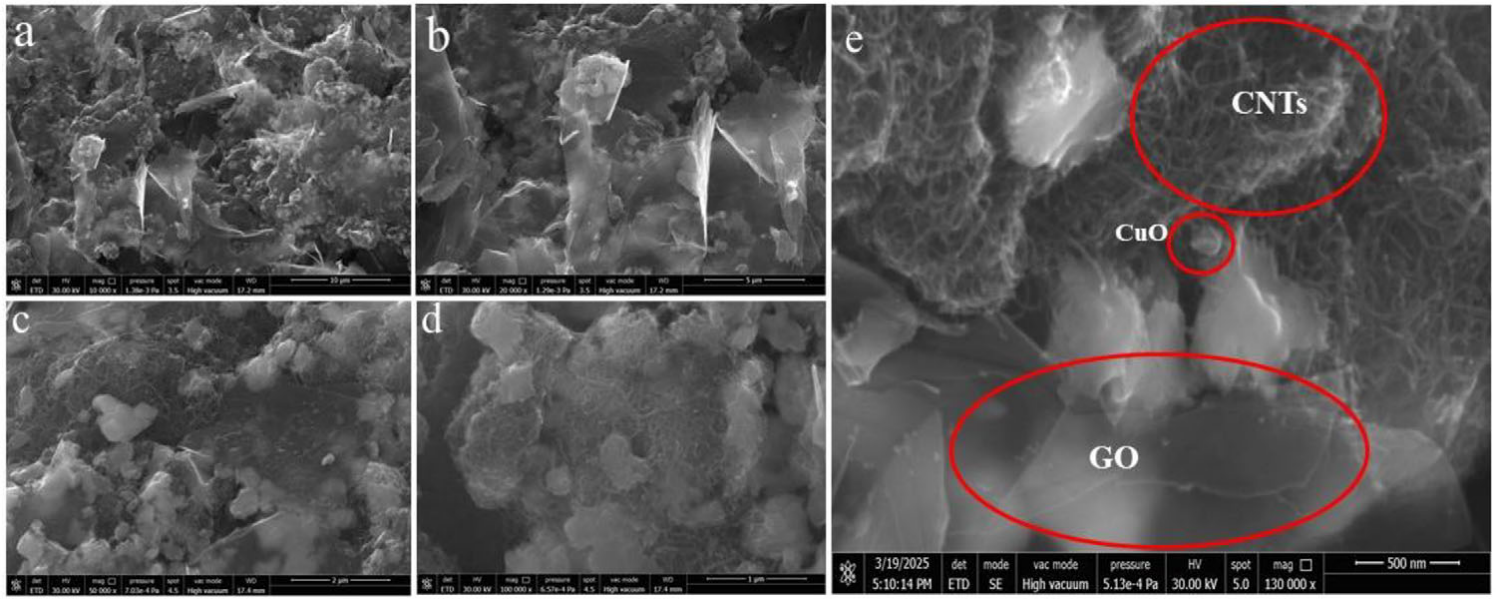
SEM images of nanohybrid (CuO@CNTs/GO) material with (a and b) lower and (c–e) higher magnifications.

Morphological characterization of CuO@CNTs/GO hybrid composite was performed by scanning electron microscopy (SEM), which represents the morphology of as‐prepared composite for glucose sensing. Figure 2a,b illustrates CuO NPs covering the net‐like CNTs and graphene sheets appearing as a thin wrapper at lower magnification. Higher magnification SEM images (Figure 2c,d) show densely populated CuO nanoparticle clusters embedded in a composite to enhance mechanical strength and electrochemical properties owing to the synergistic effect of metal NPs with carbon material. High‐resolution SEM (500 nm) clearly confirms the presence of CNTs as shown in Figure 2e. The SEM image of multi‐walled CNTs typically confirms entangled and tube‐like structures that indicate their fibrous nature. Incorporation of CNTs can limit the restacking of graphene sheets, whereas the presence of graphene can ensure uniform distribution of CNTs. This may increase the total exposed surface area, consequently boosting the electrocatalytic activity of nanohybrid (CuO@CNTs/GO), which would be advantageous for biosensing applications.

**FIGURE 3:**
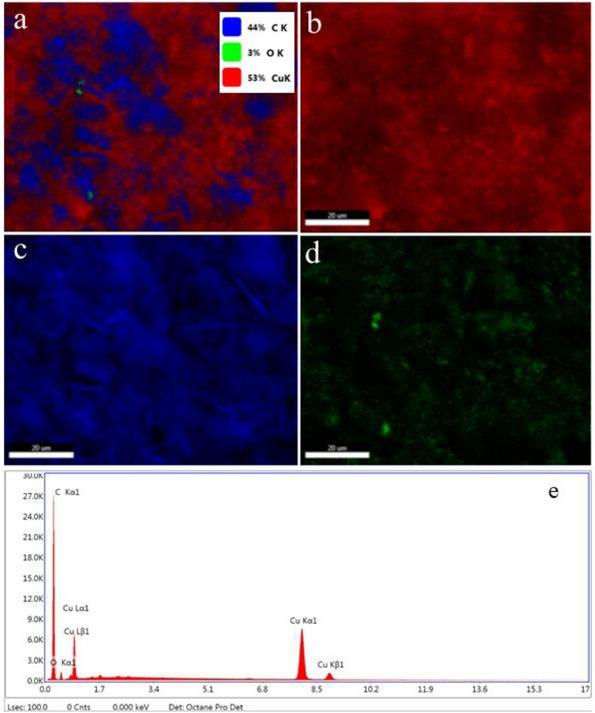
Elemental mapping images of nanohybrid (CuO@CNTs/GO) with (a) survey analysis, (b) copper, (c) carbon, (d) oxygen and (e) elemental abundance ratio in nanohybrid.

The elemental composition of the prepared nanohybrid is represented by energy‐dispersive x‐ray (EDX) spectroscopy as shown in Figure 3. EDX spectrum indicated the peaks corresponding to copper, carbon and oxygen, which shows that the compound contains respective elements of the components only, thus confirming the purity and presence of the necessary elements. Figure 3a–d represents the results of elemental mapping indicating the presence of copper, oxygen and carbon constituents. These images confirm that the synthesized hybrid composite possesses high purity and homogeneous distribution of component elements. The deepest colour revealed the highest carbon existence, which is also confirmed by the relative abundance ratio peak of the elements given in Figure 3e.

### 2.2 Electrochemical Studies

**FIGURE 4:**
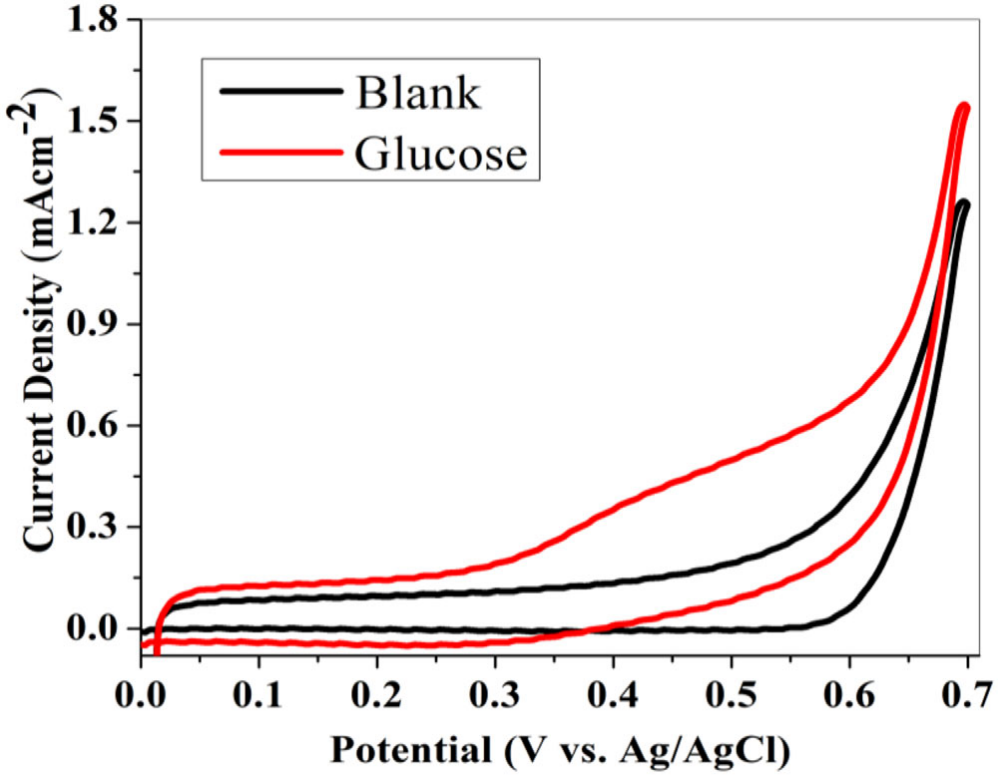
Cyclic voltammogram of nanohybrid (CuO@CNTs/GO) modified electrode in the absence and presence (0.74 mM) of glucose under alkaline conditions with fixed scan rate of 50 mV/s.

The electrochemical performance of fabricated electrode composed of nanohybrid (CuO@CNTs/GO) modified CNTs fibre was examined by cyclic voltammetry in a potential window of 0–0.7 V versus Ag/AgCl under alkaline conditions. The effective surface area of the modified electrode (CuO@CNTs/GO) was determined to be 0.0235 cm^2^, providing a well‐defined interface for electrochemical reactions. This optimized surface enhances the catalytic performance and charge transfer efficiency. Cyclic voltammogram is a plot of current versus applied potential for the response of electrooxidation of glucose. This graph provides valuable information about the electrochemical behaviour of the analyte, the mechanism of electron transfer, thermodynamics and kinetics of redox reaction. Figure 4 represents the cyclic voltammogram of the fabricated electrode in a blank solution and the presence of 0.74 mM glucose. A prominent oxidation peak is formed at a potential of ∼0.5 V with a reasonable current density of 0.5 mA cm^−2^, showing excellent electrocatalytic activity of synthesized nanohybrid for glucose oxidation. Cathodic peak was not observed in the reverse scan, as the glucose oxidation reaction is irreversible at the working electrode surface. The faradaic current was initiated at 0.35 V, reaching its maximum value at 0.5 V, which can be attributed to the oxidation of glucose at the modified electrode. Moreover, the current value has shown a steady increase at 0.6 V due to the oxidation of water. The possible mechanism of glucose oxidation at the CuO‐modified electrode is shown in the following equations [38, 39]:

(1) $$\mathrm{CuO}+OH^{-}\to \mathrm{CuOOH}+e^{-}$$

(2) $$\mathrm{CuOOH}+\mathrm{glucose}+e^{-}\to \mathrm{Cu}{\left(\mathrm{OH}\right)}_{2}+\mathrm{gluconolactone}$$

In the presence of an alkaline medium, CuO or Cu^2+^ undergoes oxidation to form CuOOH or Cu^3+^, acting as an electron transfer mediator to convert glucose to gluconolactone, which accepts an electron. This electron shift from glucose to electrode causes an increase in oxidation current, resulting in an irreversible glucose oxidation process.

**FIGURE 5:**
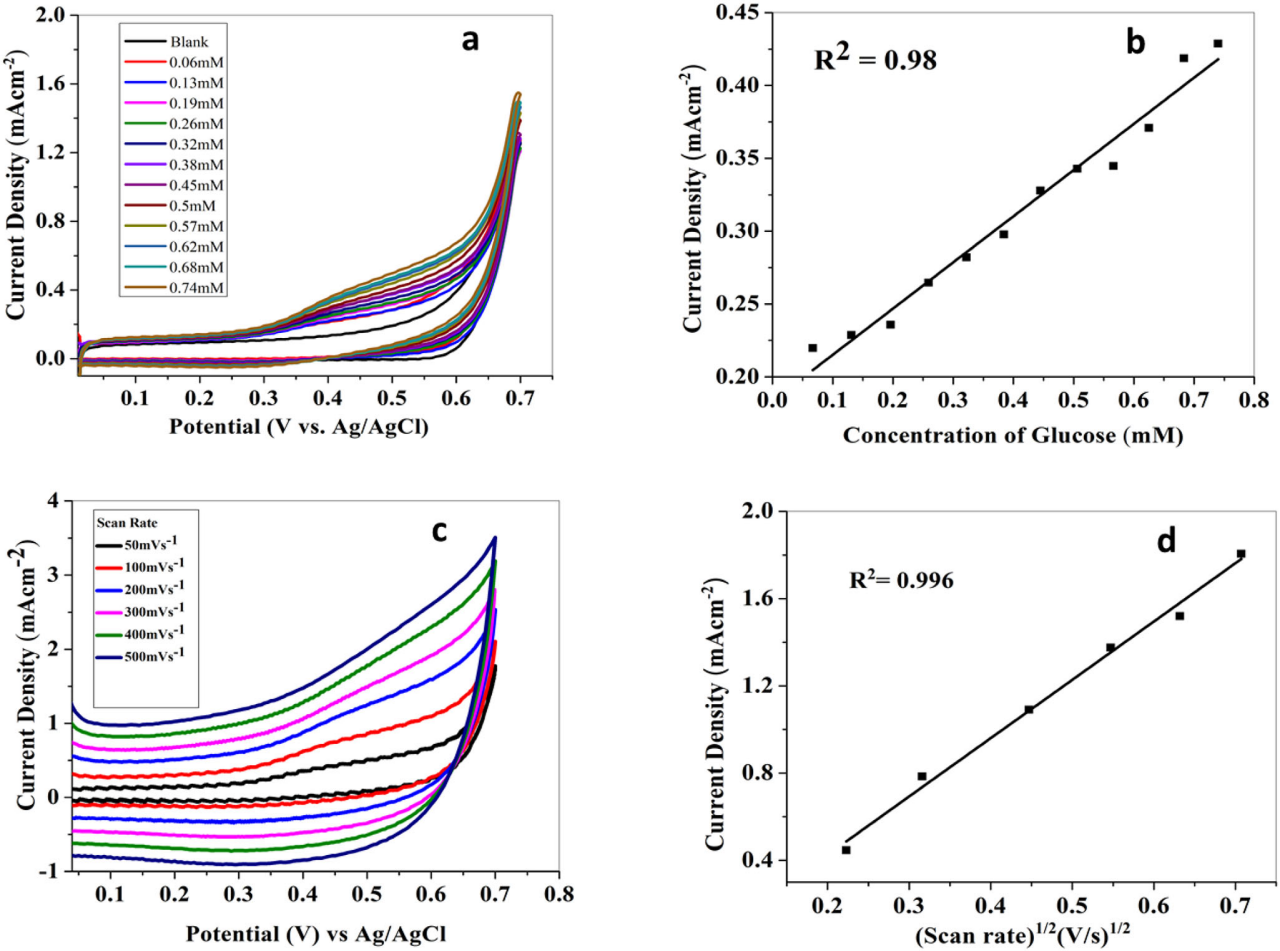
(a) Cyclic voltammograms of modified electrode at different concentrations of glucose at a fixed scan rate of 50 mV s^−1^ and (c) at different scan rates with a fixed concentration of glucose in 0.1 M KOH and their (b and d) corresponding calibration curves.

Figure 5a shows overlaid voltammograms recorded for varying glucose concentrations with a fixed scan rate of 50 mV s^−1^ in 0.1 M KOH that represents a significant increase in current density proportional to an increase in the amount of glucose with a characteristic anodic peak at 0.5 V versus Ag/AgCl. The relation of current (output) and concentration (input) provides sensitivity of the electrocatalyst, which is an important parameter indicating the performance of the biosensor. Figure 5b represents a linear relationship between current density and glucose concentration, whereas the slope of this graph represents sensitivity, which is calculated to be 328 mA cm^−2^ Mm^−1^.

Voltammograms observed at various scan rates are used to investigate and calculate kinetic parameters like rate constant, electron transfer coefficient, kinetic process and effective surface area of modified electrode. Figure 5c displays voltammograms recorded at varying scan rates ranging from 50 to 500 mV s^−1^ to study the mass transfer process at the modified electrode under the same conditions (0.74 mM glucose, 0.1 M KOH). The observed variations in carrier mobility and current density can be ascribed to the sample morphology and the influence of matrix‐induced dipole interactions at the electrode interface, as reported in similar nanostructured systems [40, 41]

$$i_{p}=2.69\times 10^{5}\times n^{3/2}AD^{1/2}Cv^{1/2}$$

With a steady increase in scan rate, higher current density values were increased along with the shifting of peak potentials to more positive potential. This is due to rapid electrolyte flux towards electrode and diffusion phenomena. Figure 5d represents a linear relation between current density and the square root of scan rate, which is an indication of diffusion‐controlled reaction at modified electrode surface as represented by the Randles–Sevcik equation:

(3) $$i_{p}=2.69\times 10^{5}\times n^{\left(3/2\right)}AD^{\left(1/2\right)}C\upsilon^{\left(1/2\right)}$$

where $i_{p}$ represents the peak current and ν is the scan rate.

### 2.3 Chronoamperometric Studies

**FIGURE 6:**
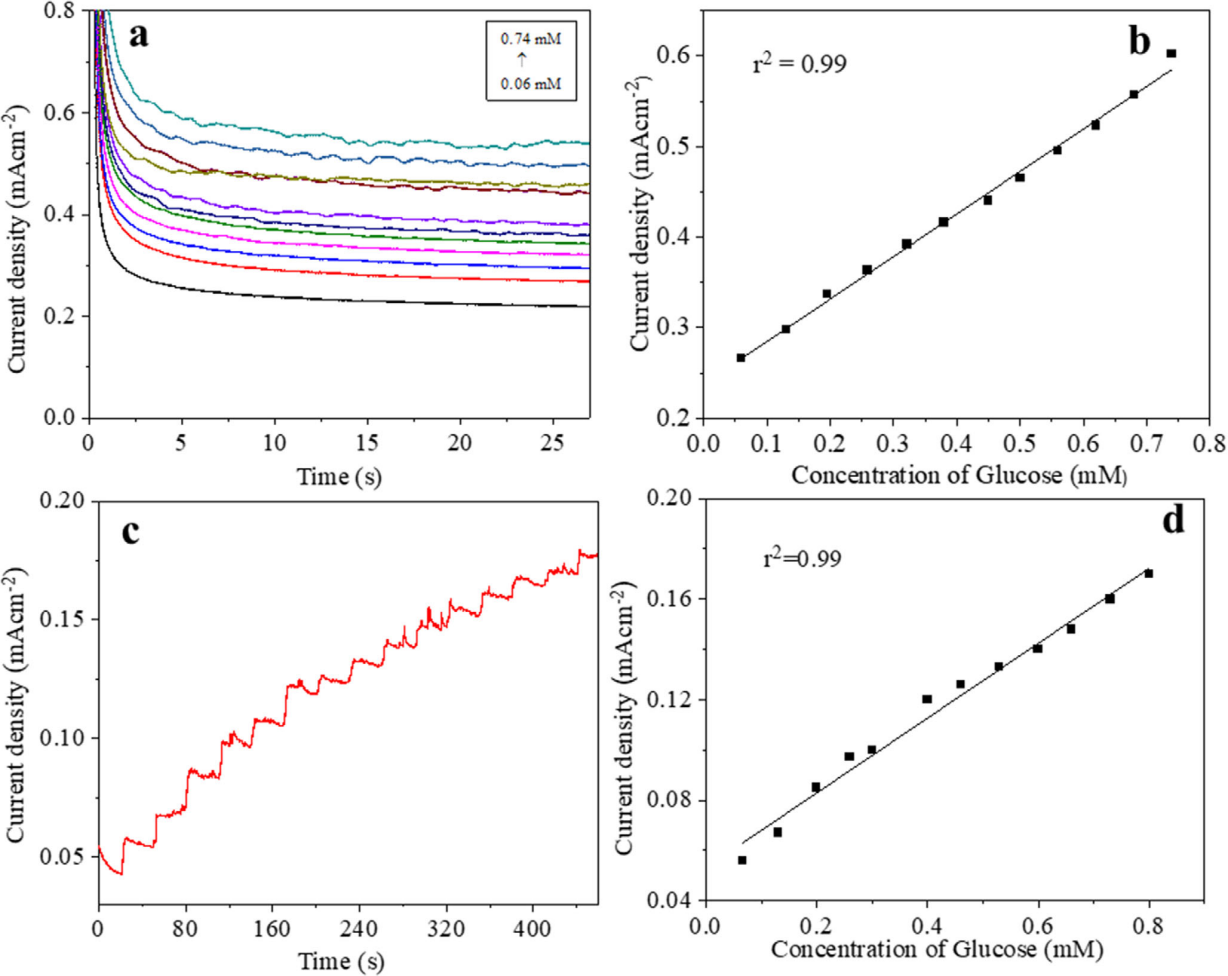
(a) Amperometric response of the fabricated electrode with the addition of glucose under stirring in 0.1 M KOH; (c) staircase amperometric response of modified fibre with successive addition of glucose under the applied potential of 0.5 V (vs Ag/AgCl) and their (b and d) corresponding calibration curve.

Chronoamperometric measurements were investigated by plotting current response versus time at fixed applied potential with the continuous addition of the analyte. These evaluations are used to calculate the detection limit, response time, linear range, long‐term performance and stability of electrochemical sensors. Figure 6a represents the electrochemical behaviour of the fabricated electrode through the chronoamperometric method. The current response was recorded for different concentrations of glucose added in 0.1 M KOH solution under constant stirring at a fixed applied potential of 0.5 V versus Ag/AgCl as a reference electrode. By using the standard deviation and slope value of the calibration curve in Figure 6d, the limit of detection was calculated to be 0.033 mM with linear response from 0.06 to 0.74 mM. The limit of detection was determined by using the following formula: limit of detection = (SNR × *σ*)/slope, where *σ* is the standard deviation of blank and slope is the calibration curve.

Electrochemical sensing ability of the modified electrode for glucose was studied by utilizing the amperometric method. Current values were recorded by successive additions of glucose solution to continuously stirred 0.1 M KOH solution in an electrochemical cell under optimized conditions. Figure 6c represents the amperometric response of glucose solution over a prolonged duration (500 s) at an applied constant potential of 0.5 V versus Ag/AgCl observed after regular intervals of 30 s. Although the corresponding calibration curve (Figure 6d) represented the two types of behaviour, at lower concentrations, it displayed the higher slope value, and in the higher concentration region, the slope value was depressed for the concentration of 0.5–0.8 mM.

**FIGURE 7:**
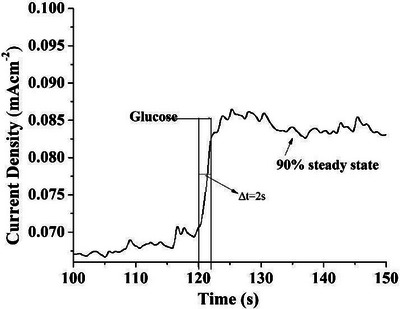
Magnifying plot of single‐step staircase chrono measurements to assess the response time under steady‐state conditions.

The as‐prepared sensor exhibited a staircase incremental behaviour of current and a very fast response to the change in glucose concentration, achieving steady‐state current within 2 s. Magnified view of the staircase response of copper oxide–modified electrode in 0.1 M KOH is shown in Figure 7. The fabricated electrode exhibited an excellent response time and wide linear range up to 0.74 mM. Such a fast response of the sensor towards oxidation of glucose is attributed to the synergistic effect of superior catalytic activity of copper oxide NPs acting as a mediator for fast electron transfer between glucose and the surface of electrode, thus promoting oxidation and excellent electrical properties of carbon‐based materials, that is, CNTs and GO.

### 2.4 Electrochemical Impedance Spectroscopy (EIS)

**FIGURE 8:**
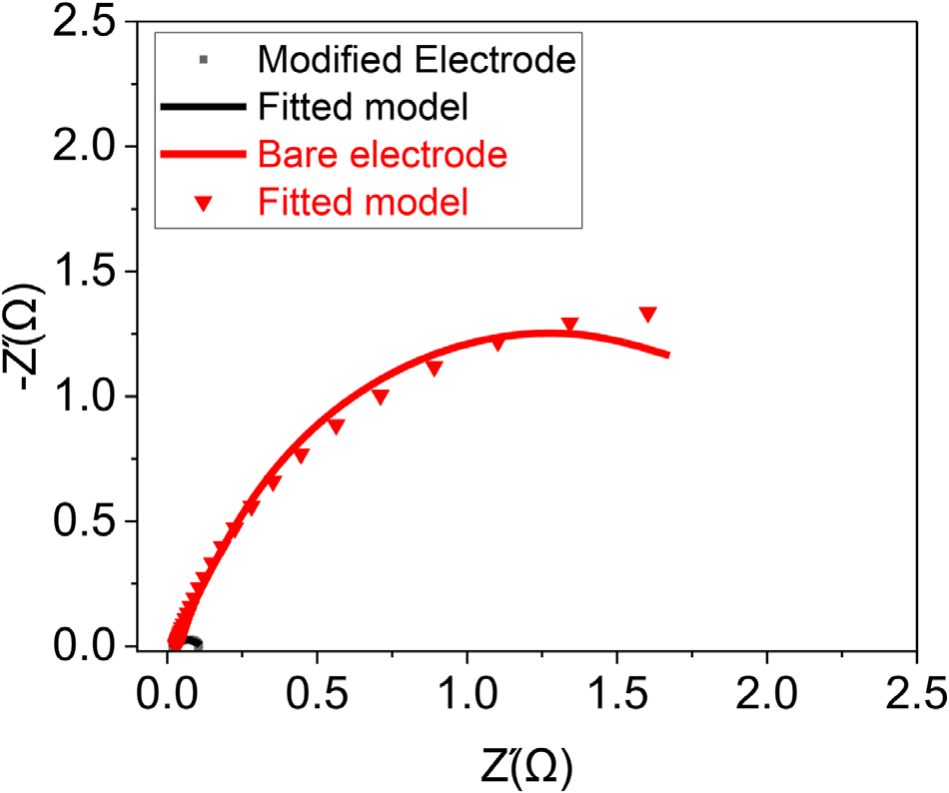
Impedance performance of the bare and modified electrodes.

EIS was employed to investigate the charge transfer kinetics and interfacial properties of the modified electrodes. Figure 8 illustrates the Randle equivalent circuit model used to analyse EIS data. The charge transfer resistance (*R*_ct_) of both modified (CuO@CNTs/GO) and bare electrodes is represented by the semicircle diameter in the Nyquist plot. A comparison between the modified electrode and the bare electrode reveals distinct semicircle diameters, indicating different *R*_ct_ values. Specifically, CuO@CNTs/GO exhibits a lower R_ct_ (95.6 Ω) than bare electrode (3.4 kΩ), suggesting enhanced charge transfer efficiency due to the nanocomposite. This reduction in *R*_ct_ (as shown in Table 1) confirms improved electron conductivity at the electrode surface. The CuO@CNTs/GO modified electrode exhibits lower solution resistance (*R_s_*) than the bare electrode due to enhanced conductivity from the CNTs/GO network and improved charge transfer via CuO NPs.

**TABLE 1:** Charge transfer resistance (*R*_ct_) and solution resistance (*R_s_*) derived from electrochemical impedance spectroscopy (EIS) spectra.

Electrode	R_s_ (Ω)	R_ct_ (Ω)
Modified	16.78	95.6
Bare	30	3441

This confirms the superior electrochemical performance of the fabricated composite over the pristine electrode. These EIS findings demonstrate that CuO@CNTs/GO exhibits superior electron conductivity and reduced charge transfer resistance compared to bare electrode.

### 2.5 Interference Study

**FIGURE 9:**
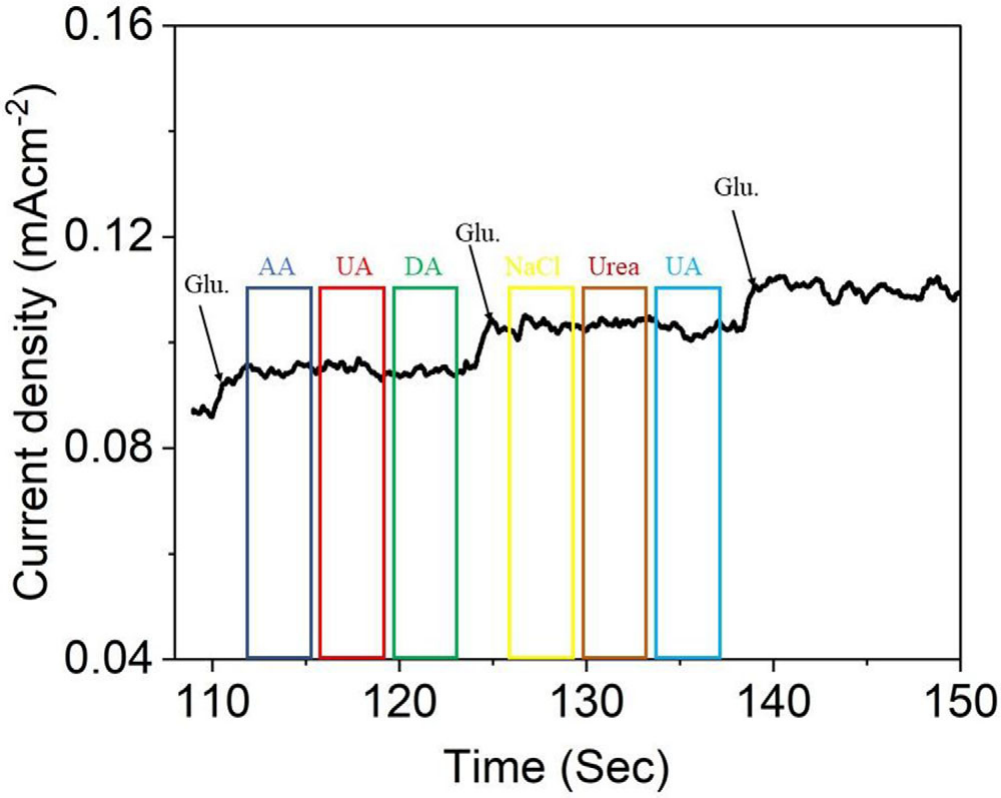
Interference study of the sensor in the presence of common interferents. AA, ascorbic acid; DA, dopamine; UA, uric acid.

Moreover, the selective sensing performance of CuO@CNTs/GO towards glucose was investigated via chronoamperometry at 0.45 V in the presence of potential interfering analytes, namely, AA, uric acid (UA), dopamine (DA), NaCl and urea. As shown in Figure 9, the measured peak currents for these interfering species were significantly lower compared to that of glucose. This marked difference in current response underscores the high selectivity of the CuO@CNTs/GO sensor for glucose detection over other common biomolecules.

### 2.6 Stability and Reusability

**FIGURE 10:**
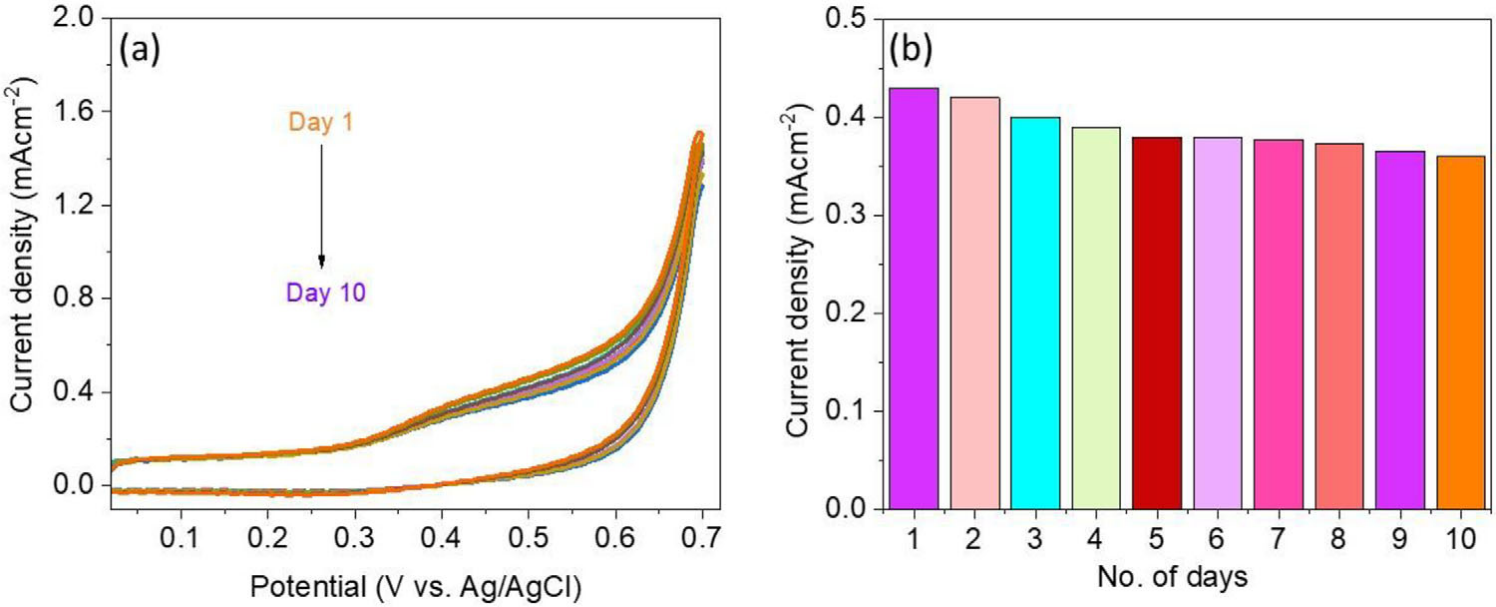
(a) CV cycles of CuO@CNTs/GO for 10 days; (b) bar graph of current obtained for number of days.

Stability and reusability are crucial features of the electrochemical sensor. In this study, the stability as well as the reusability of the sensor was assessed by storing it for 10 days and subsequently testing it. The voltammetric response of the previously fabricated electrodes closely matched its initial performance, retaining 97% of the original current response (given in Figure 10) thereby confirming its exceptional stability and reusability.

Table 2 provides information of the electrode materials reported so far, used for the detection of glucose utilizing the electrochemical sensing approach.

**TABLE 2:** Comparison of selected electrode material for the detection of glucose.

Electrode material	Analyte	Detection range (µM)	Limit of detection (µM)	Refs.
Nf/Au‐CuO/GCE	Glucose	5–650	1.4	[42]
Co_3_O_4_/CNTs	Glucose	4–4.74 mM	2	[43]
CuO@MCM‐41	Glucose	83–1500	0.016	[44]
Cu/g‐SiCNT/CuO	Glucose	1–4480	0.8	[45]
GO/MWCNT/Au@Pt/GCE	Glucose	Up to 100	0.042	[46]
Cu–CuO/C	Glucose	Up to 3 mM	5	[47]
CuO@CNTs/GO	Glucose	60–74	33	This work

Abbreviations: CNT, carbon nanotube; GCE, glassy carbon electrode; GO, graphene oxide.

## 3 Conclusion

A nanohybrid‐modified fibre (CuO@CNTs/GO) electrode was successfully fabricated to develop a microelectrode for electrochemical nonenzymatic glucose sensing. This unique hybrid composite displayed higher electrochemical performance than single‐component CNTs or graphene electrodes. Electrocatalytic oxidation of glucose by the developed electrode was found to be very efficient with a high sensitivity of 328 mA mM^−1^ cm^−2^ and a low detection limit of 0.033 mM with a wide linear range. Hence, the outcomes indicate that CuO@CNTs/GO could be a promising material to fabricate a flexible microelectrode for the efficient nonenzymatic detection of glucose.

## Author Contribution

**Amina Khalid**: investigation, data curation, writing–original draft. **Rizwan Shoukat, Abid Ali, Aarfa Sajid, Amel Y. Ahmed**: conceptualization, supervision, methodology, project administration, resources, funding acquisition. **Qaisar Manzoor, Salih Akyürekli**: methodology, validation, software. **Muhammad Adeel Asghar**, **Rizwan Shoukat**, **Arif Nazir**, **Norah Alsadun**: validation, writing–review and editing.

## Conflicts of Interest

The authors declare no conflicts of interest.

## Data Availability

All data generated or analysed during this study are included in this article.
